# Supplementary material for: What does it take to learn the rules of RNA base pairing? A lot less than you may think
Source: bioRxiv. 2025 Aug 2:2025.07.31.668042. Preprint. [Version 1] doi: 10.1101/2025.07.31.668042 (PMC12324431; doi:10.1101/2025.07.31.668042)
Supplement: Supplement 1 [file media-1.gz › supplemental_material/src/rscape_v2.6.0.tar.gz!/rscape_v2.6.0/lib/infernal/Manuscripts/banded-cyk/figs/speedup.pdf]

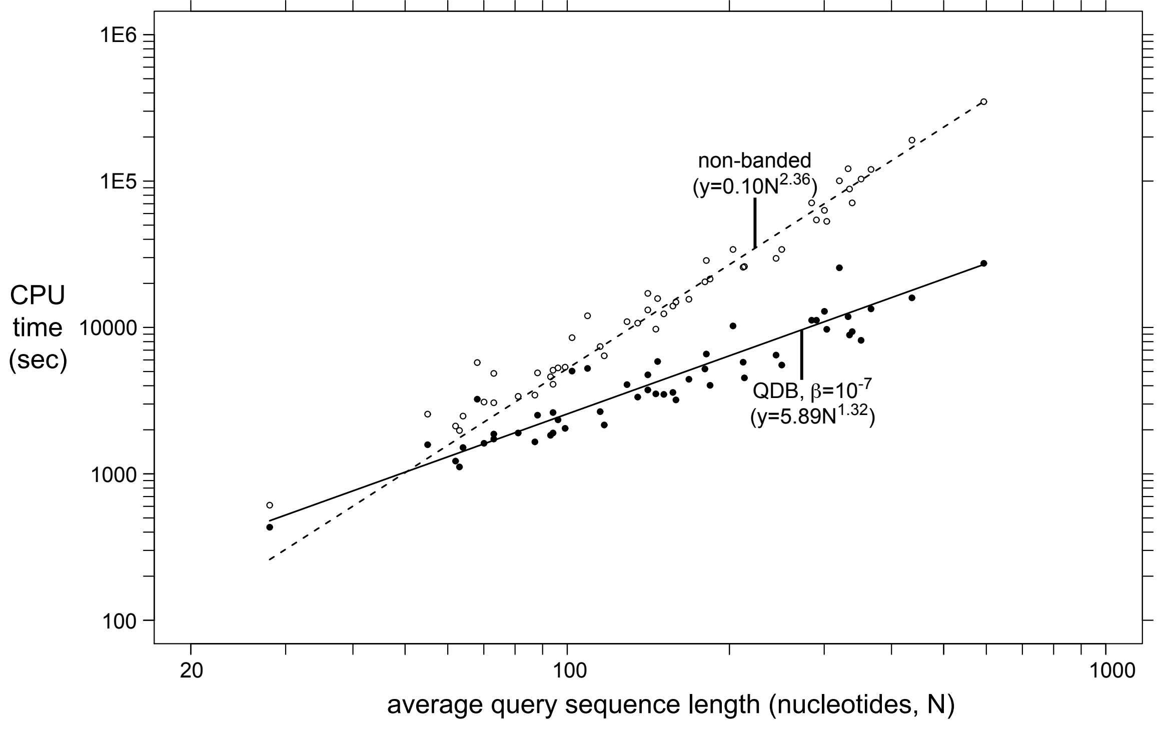

CPU time (sec)
1E6
1E5
10000
1000
100
20
100
1000
average query sequence length (nucleotides, N)
non-banded
(y=0.10N^2.36)
QDB, β=10^-7
(y=5.89N^1.32)
